# A Short Evidence‐Based, Belief‐Focused Educational Video Improves Back Pain Beliefs but Not Lifting Behaviour in Healthy Office Workers: An Exploratory Randomised Controlled Trial

**DOI:** 10.1002/msc.70290

**Published:** 2026-10-23

**Authors:** Anja Müller‐Schmid, Christian Bangerter, Panka Nagy, Fabian Pfeiffer, Marina Bruderer‐Hofstetter, Guillaume Christe, Stefan Schmid

**Affiliations:** ^1^ School of Health Professions Division of Physiotherapy Bern University of Applied Sciences Bern Switzerland; ^2^ School of Health Sciences Institute of Physiotherapy ZHAW Zurich University of Applied Sciences Winterthur Switzerland; ^3^ School of Health Professions Institute on Ageing Bern University of Applied Sciences Bern Switzerland; ^4^ HESAV School of Health Sciences‐Vaud HES‐SO University of Applied Sciences and Arts Western Switzerland Lausanne Switzerland

**Keywords:** lumbar spine, motor behaviour, movement strategy, spinal motion

## Abstract

**Background:**

Unhelpful beliefs about low back pain are associated with altered movement behaviour during lifting. Brief educational videos may offer a scalable approach to modify such beliefs, but their impact on movement behaviour remains unclear.

**Objective:**

To investigate whether a short educational video can reduce unhelpful back pain beliefs and promote a more stoop‐like lifting behaviour in healthy adults.

**Methods:**

In this assessor‐blind randomised controlled trial, 50 healthy office workers were assigned to an experimental group (educational video on back pain beliefs and management) or a control group (anatomy video). Outcomes were measured before and immediately after the intervention and included the 10‐item Back Pain Attitudes Questionnaire (Back‐PAQ10), lumbar curvature range of motion (RoM), and lifting strategy (Stoop‐Squat Index) during lifting and lowering of 5 and 15 kg boxes. Linear mixed‐effects models evaluated Group, Time, and interaction effects.

**Results:**

Back‐PAQ10 scores showed a larger reduction in the intervention group (Δ = 6.12, *p* < 0.001) than the control group (Δ = 0.88, *p* = 0.002), with a significant Group × Time interaction (*p* < 0.001). Lumbar curvature RoM decreased (*p* < 0.001), but no Group × Time interaction occurred (*p* = 0.604). The Stoop‐Squat Index showed a main effect for Time (*p* = 0.005), but no Group × Time interaction (*p* = 0.935).

**Conclusion:**

A brief educational video effectively improved back pain beliefs but did not alter lifting behaviour or spinal motion. This dissociation between cognitive and motor responses suggests that changing movement patterns may require more intensive or interactive interventions.

## 1 Introduction

Low back pain (LBP) is one of the most common musculoskeletal disorders, with nearly seven out of ten people in Switzerland experiencing it regularly (Mueller 2021). Although relatively rare in childhood, its prevalence increases sharply during adolescence, and individuals with a history of LBP are at higher risk of recurrent episodes (Hartvigsen et al. 2018). Despite substantial healthcare spending on diagnostics and treatment (Deyo et al. 2009), most cases of LBP resolve spontaneously within 6–12 weeks (Maher et al. 2017; National Guideline Centre (UK) 2016), and fewer than 5% require medical or surgical intervention (Hartvigsen et al. 2018; O’Sullivan and Lin 2014). Nevertheless, LBP can negatively affect daily activities and quality of life in 20%–30% of sufferers (O’Sullivan 2012), contributing to persistent beliefs that the back is fragile and must be protected (Wernli et al. 2022).

Such beliefs influence movement, particularly during lifting, as pain‐related fear has been associated with reduced lumbar motion even in asymptomatic individuals (Bangerter et al. 2025; Knechtle et al. 2020). However, evidence suggests that regular bending or maintaining a flexed lumbar spine is not associated with the development or persistence of LBP, even in activities such as rowing or cycling (Belavy et al. 2019; Foss et al. 2012; Lagersted‐Olsen et al. 2016; Maillard et al. 2026; Saraceni et al. 2020; Wernli et al. 2022). Moreover, discontinuing protective behaviours has been associated with reduced pain and greater confidence in movement (Nzamba et al. 2024; Wernli et al. 2022). This aligns with recent findings showing that individuals with chronic LBP who adopt stoop‐like or faster lifting patterns often experience improvements in pain and function, challenging traditional ergonomic prescriptions that favour ‘safe’ squat techniques (Au et al. 2025).

In addition to biomechanical factors, cognitive and emotional factors play a key role in shaping movement and back‐related beliefs. Fear, worry, and other negative emotions can restrict movement patterns and reinforce the perception of spinal vulnerability (Nolan et al. 2018; Wernli et al. 2022). These beliefs are often reinforced by personal pain experiences, societal norms, and guidance from healthcare professionals (Chang et al. 2026; Christe et al. 2021; Darlow 2016; Wernli et al. 2022).

Educational interventions, including websites and videos, have been developed to counteract unhelpful beliefs about the spine (Corbat et al. 2020). With social media playing an increasingly central role in information dissemination (O’Keeffe et al. 2019), brief educational videos may be a practical tool for promoting evidence‐based understanding.

The present study aims to explore, in healthy participants, whether a short educational video on the current evidence of back beliefs and LBP prevention may reduce unhelpful perceptions and promote more stoop‐like lifting behaviour (i.e., greater lumbar flexion and less squatting) compared to a control video presenting general back anatomy.

## 2 Methods

### 2.1 Study Design

This study was designed as an assessor‐blind, two‐arm parallel‐group randomised controlled trial with a 1:1 allocation ratio. Participants were randomly assigned to either an experimental group receiving an educational video on the current evidence of LBP prevention and back beliefs or a control group viewing a video on general back anatomy. Outcome assessors were blinded to group allocation, with participants wearing headphones and outcome assessors leaving the room during the intervention. Although full participant blinding was not feasible, efforts were made to blind participants as much as possible by not explicitly informing them about their group allocation. Outcomes were assessed before and immediately after the intervention. The study protocol was approved by the local ethics committee (KEK Bern, no. 2023–01182), and all participants provided written informed consent. The trial was prospectively registered at the German Clinical Trials Register (ID: DRKS00032348; https://drks.de/search/en/trial/DRKS00032348/details; registered on 24 July 2023). This study was reported in accordance with the CONSORT guidelines.

### 2.2 Participants and Randomisation

Participants were recruited via email‐distributed flyers among office workers of a Swiss company. Recruitment and data collection took place in November 2023. Potential candidates were included if they met the following criteria: aged between 18 and 65 years, oral and written proficiency in the German language, no back pain episode in the last 3 months, no diseases, comorbidities, injuries, surgeries or pain restricting the ability to lift, no history of chronic LBP (pain lasting more than 3 months), spinal disorders or surgery, no known pregnancy or breastfeeding, and a body mass index (BMI) < 30 kg/m^2^. Because this was an exploratory trial intended to provide preliminary estimates of intervention effects, the sample size was determined by feasibility rather than a formal power calculation. A sample of 50 participants was considered feasible within the available resources and timeframe. Participants were randomly assigned to either the experimental or control group in a 1:1 ratio using a sealed‐envelope procedure. Specifically, 50 envelopes were prepared, each containing two differently coloured paper slips indicating group allocation and the starting weight for the lifting task, ensuring concealed and unbiased assignment.

### 2.3 Interventions

#### 2.3.1 Evidence‐Based, Belief‐Focused Education

Participants in the experimental group watched an educational video of approximately 3 min (Link to video here) presenting evidence‐based information on LBP prevention and management, with a particular focus on unhelpful back beliefs, in plain language. In the video, healthcare professionals (i.e., a psychologist, a physician, and a physiotherapist) explain how common beliefs about the back being fragile or easily injured can influence LBP experiences. A patient also shared personal experiences managing LBP. The video emphasised self‐management, gradual movement, and building confidence in using the back during everyday activities. It did not prescribe, demonstrate, or provide instruction in a specific lifting technique. The content was adapted from a French educational website (infomaldedos.ch) and translated into German, with prior review and feedback from LBP experts, patients, and healthcare professionals (Corbat et al. 2020). English subtitles for the video are available as SRT and VTT files in the electronic supplementary materials.

#### 2.3.2 Structure‐Focused Education

The control group viewed a video of approximately 2 min on general back anatomy (Link to video here), describing the spine's structure, intervertebral discs, and supporting muscles and ligaments. This video did not provide guidance on LBP management or challenge beliefs about back vulnerability and served to control for attention and exposure to video content.

### 2.4 Assessments and Outcomes

Assessments at baseline (Pre) and immediately following the intervention (Post) were conducted on site in a designated closed room at the participating company in Switzerland. They included a questionnaire on back beliefs, as well as measurements of lumbar spine motion and lifting strategy during object handling. All study procedures were conducted within a single visit by the same examiner, an experienced physiotherapist, to ensure consistency. Before the baseline assessment, participants' demographics, including age, mass, height, and sex, were recorded, along with fingertip‐to‐floor distance (FFD) as a combined measure of back and hamstring flexibility (Perret et al. 2001), where smaller distances indicate greater flexibility, with 0 cm representing maximum flexibility.

#### 2.4.1 Back Beliefs

Participants completed the German version of the 10‐item Back Pain Attitudes Questionnaire (Back‐PAQ10), a shortened form of the original 34‐item questionnaire developed by Darlow et al. (2014). The questionnaire covers five domains: perceived vulnerability of the back (items 1–2), the link between back pain and injury (items 3–4), engagement in activities during episodes of back pain (items 5–6), psychological factors influencing back pain (items 7–8), and expected outcomes or prognosis of back pain (items 9–10) (Pierobon and Darlow 2023). Responses are given on a 5‐point Likert scale ranging from ‘False’ to ‘True,’ with intermediate options labelled ‘Possibly False,’ ‘Unsure,’ and ‘Possibly True.’ For scoring purposes, items 6, 7, and 8 are reverse‐coded. The Back‐PAQ10 total score, ranging from 10 to 50, was used as an outcome measure for back beliefs in the current study, with higher scores indicating more unhelpful back beliefs.

The German version underwent cross‐cultural adaptation and evaluation of its reliability and construct validity before its use in the present study, although one of the corresponding manuscripts was still under peer review at the time of acceptance (Nyfeler et al. 2026; Mohler et al., Under Review). The German Back‐PAQ10 total score demonstrated good test‐retest reliability for the total score (intraclass correlation coefficient = 0.905, 95% CI 0.865–0.934), with a standard error of measurement of 2.15 points on the 10–50 total‐score scale. Its internal consistency was also good (Cronbach's *α* = 0.831, 95% CI 0.780–0.874) (Nyfeler et al. 2026).

#### 2.4.2 Lumbar Spine Motion and Lifting Strategy

Participants performed five repetitions of symmetric lifting and lowering of both a 5 kg and a 15 kg box (40 × 20 × 10 cm) in randomised order and at their own pace, using a freestyle technique (i.e., no specific instructions were given). Each repetition consisted of lifting the box from the floor to upright standing, placing it back down on the floor, and returning without the box to an upright stance, while keeping the feet in the same position during all five repetitions.

Lumbar spine motion was measured using the Epionics SPINE System (MCG Motion Capture GmbH, Heidelberg, Germany), following the procedure described by Suter et al. (2020). The system comprises two flexible strain‐gauge sensor strips, which were attached to the skin using concave elastic tapes and positioned 3 cm lateral to the midline of the spine, starting at the level of the posterior superior iliac spine (Figure 1A). Sensors were connected to a portable storage unit worn around the hips with a belt.

**FIGURE 1:**
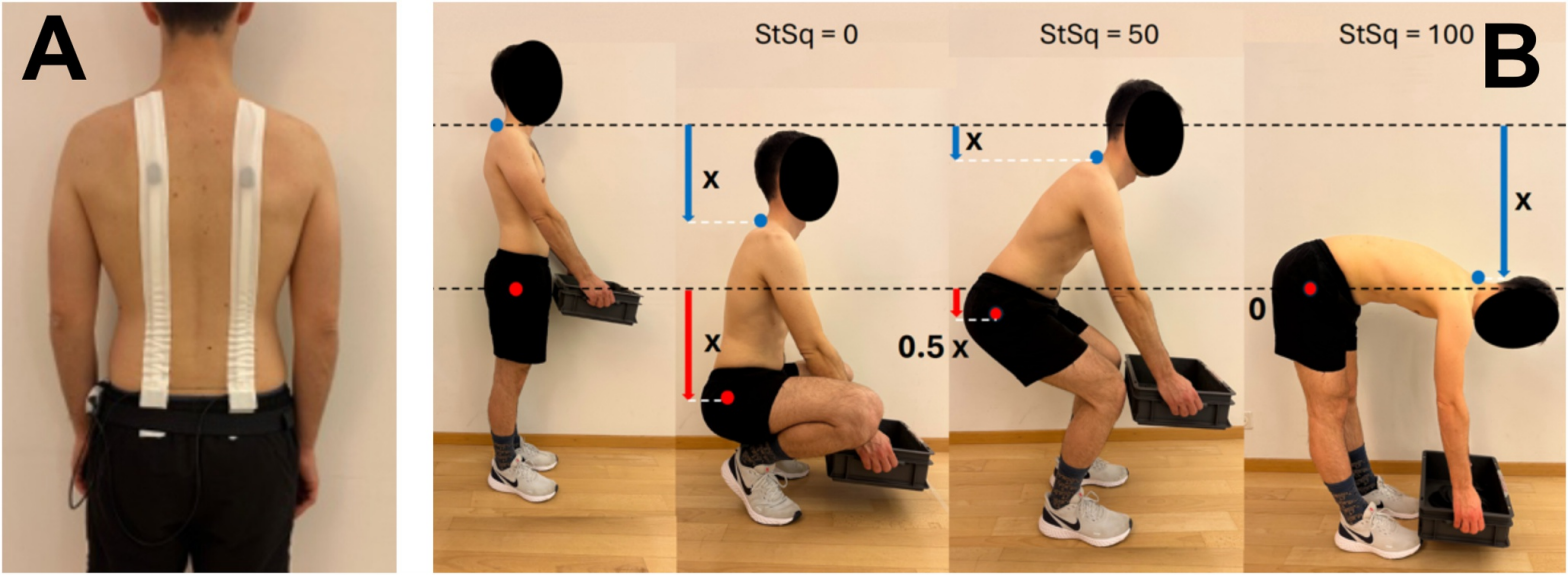
(A) Application of the Epionics SPINE system on the participants' backs. (B) Illustration of the Stoop‐Squat Index (StSq), where 0 represents a full squat and 100 represents a full stoop lift. The blue and red dots indicate the locations of the C7 spinous process and the greater trochanter, respectively.

Lumbar curvature angles were calculated as the average of the left and right sensor strips. The signals were low‐pass filtered at 6 Hz using a fourth‐order, zero‐phase Butterworth filter. Key phases for analysis were defined as lifting (from box lift‐off to reaching an upright position) and lowering (from upright position to the box touching the floor). Lumbar curvature range of motion (RoM) was used as the outcome variable for lumbar spine motion and was calculated as the difference between the maximum and minimum curvature angle for each lifting and lowering phase in each box weight condition, and then averaged across the five repetitions.

Lifting strategy was assessed using the Stoop‐Squat Index (Schmid 2022), derived from lateral‐view video recordings obtained with an iPad camera (Apple Inc., Cupertino, CA, USA; 60 Hz). The camera was mounted on a tripod at a height of 85 cm and positioned 3 m from the participant (Bangerter et al. 2024). To improve the accuracy of the Stoop‐Squat Index, reflective markers were placed on the C7 spinous process and the greater trochanter.

Video data were analysed using the Dartfish software (Dartfish, Freiburg, Switzerland) following the protocol described by Bangerter et al. (2024). For each weight condition, the marker positions were first manually identified in their initial locations and then tracked using Dartfish's automatic tracking feature. The Stoop‐Squat Index was calculated for the lifting and lowering phases of each weight condition based on the vertical positions of the markers on the C7 spinous process and the greater trochanter (Figure 1B), with 0 representing a full squat and 100 representing a full stoop. As the outcome variable for lifting strategy, the Stoop‐Squat Index was extracted at 30% of the lifting phase and 70% of the lowering phase for both box weight conditions (5 and 15 kg), and then averaged across the five repetitions.

### 2.5 Statistical Analysis

All statistical analyses were performed in R (version 4.5.1; R Core Team, Vienna, Austria), with statistical significance set at *p* ≤ 0.05. Participant characteristics at baseline were compared between the experimental and control groups using Welch's two‐sample t‐tests for the variables Age, Mass, Height, and FFD, and Fisher's exact test for Sex distribution.

Effects of the interventions on the outcome variables were analysed using three separate linear mixed‐effects models fitted with the *lme4* package (version 1.1.37). *p*‐values were calculated using Satterthwaite's approximation for degrees of freedom with the *lmerTest* package (version 3.1.3).

For back beliefs (Back‐PAQ10), a linear mixed‐effects model was specified with fixed effects for Group (structure‐focused vs. evidence‐based, belief‐focused education), Time (before vs. after the intervention), and their interaction, while controlling for Sex, Age, Mass, and Height. A random intercept for each subject accounted for repeated measurements. Type III analysis of variance (ANOVA) was used to assess the main effects and the Group × Time interaction. Marginal means were estimated to evaluate pre‐to‐post changes within each group and differences between groups at each time point.

For lumbar spine motion (lumbar curvature RoM) and lifting strategy (Stoop‐Squat Index), models included fixed effects for Group × (Time + Task + Load) and the three‐way interactions Group × Time × Task and Group × Time × Load. Group denotes intervention assignment (structure‐focused vs. evidence‐based, belief‐focused education), Task denotes movement direction (lifting vs. lowering), and Load denotes the box weight (5 vs. 15 kg). Covariates included Sex, Age, Mass, and Height. Random intercepts were included for Subject, Subject × Task and Subject × Load to account for repeated measures across tasks and loads. Type III ANOVAs were conducted to assess the main effects and interactions. Estimated marginal means and pairwise comparisons were calculated to examine (1) changes over time within each group, (2) group differences at each time point, and (3) time effects within combinations of Group and Task as well as Group and Load.

### 2.6 Generative Artificial Intelligence (AI)

ChatGPT (OpenAI, GPT‐5.5) was used solely to assist with language editing and improvement of text clarity during manuscript preparation. No artificial intelligence tools were used to generate, analyse, or interpret scientific data. All scientific content, analyses, and conclusions were developed and verified by the authors.

## 3 Results

### 3.1 Participants

All 50 included participants—25 allocated to the experimental group and 25 to the control group—completed the study procedures and were included in the analysis (Table 1, Figure 2). There were no missing data. Baseline characteristics were comparable between the groups, with no statistically significant differences in Age, Mass, Height, Sex distribution, or FFD.

**TABLE 1:** Participant characteristics at baseline for the total sample and by group (experimental vs. control).

	Total (*n* = 50)	Experimental group (*n* = 25)	Control group (*n* = 25)	Group diff. (*p*‐values)
Age [years]	39.7 ± 10.5	37.6 ± 9.2	41.7 ± 11.5	0.168
Mass [kg]	77.6 ± 10.8	77.5 ± 11.0	77.7 ± 10.8	0.389
Height [cm]	177.5 ± 6.8	177.1 ± 7.4	177.9 ± 6.3	0.832
Sex [m/f]	40/10	21/4	19/6	0.725
FFD [cm]	5.9 ± 8.6	4.2 ± 7.9	7.6 ± 9.1	0.162

*Note:* Between‐group differences were assessed using Welch's two‐sample t‐tests for continuous variables and Fisher's exact test for the categorical variable.

Abbreviation: FFD, fingertip‐to‐floor distance.

**FIGURE 2:**
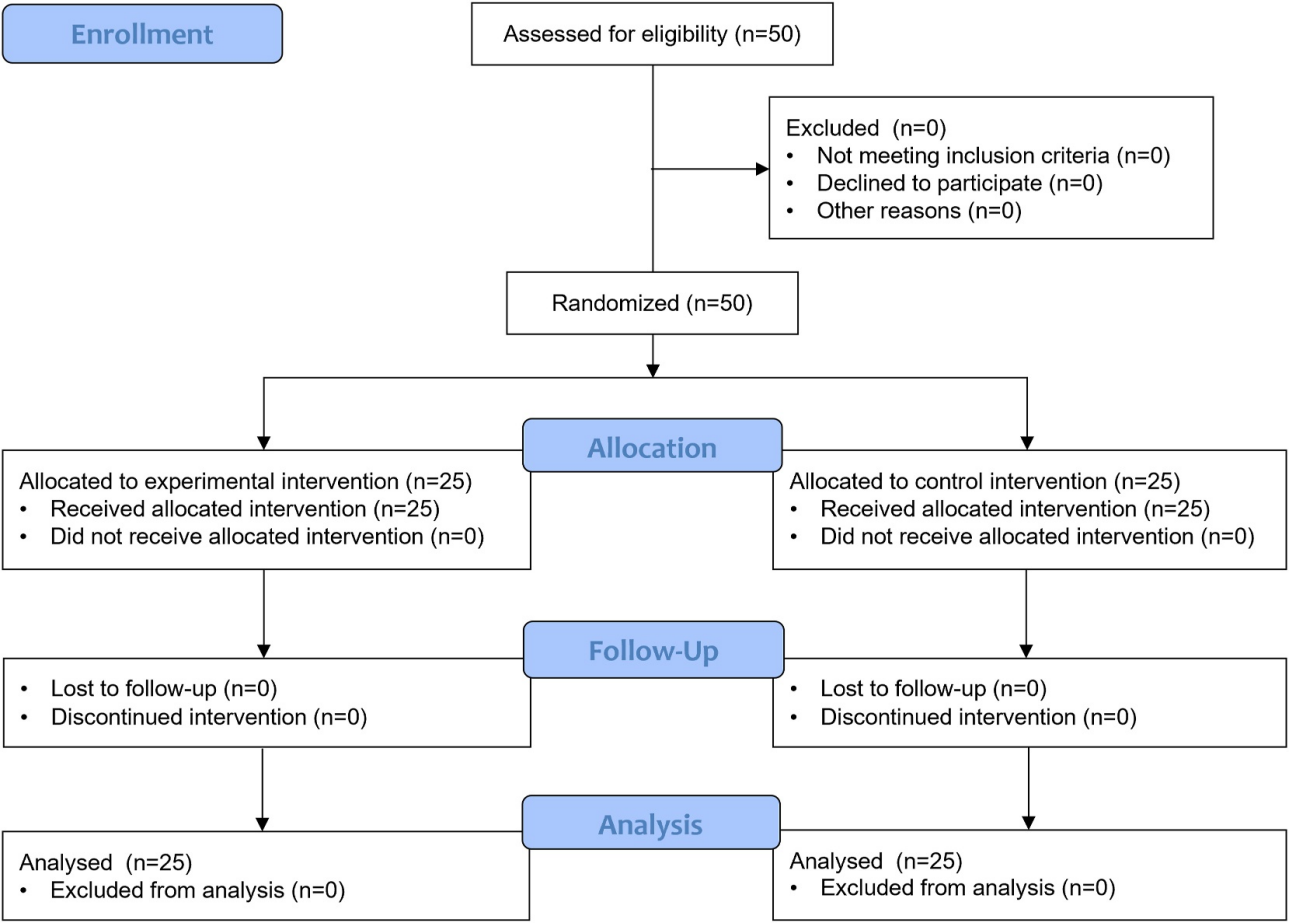
Participant flow diagram according to the CONSORT 2025 Statement.

### 3.2 Effects of Experimental Intervention

#### 3.2.1 Back Beliefs

The mixed‐effects model revealed a significant Group × Time interaction (*p* < 0.001), indicating that changes in Back‐PAQ10 over time differed between the experimental and control groups (Table 2). There were also significant main effects of Time (*p* < 0.001) and Group (*p* = 0.022). Estimated marginal means indicated that Back‐PAQ10 decreased significantly from Pre to Post in both groups, with a markedly larger reduction in the experimental group (Experimental: *Δ* = 6.12, *p* < 0.001; Control: *Δ* = 0.88, *p* = 0.002) (Figure 3). Group comparisons at each time point showed no significant difference at pre‐intervention (*p* = 0.442), but a significant difference at post‐intervention (*p* < 0.001).

**TABLE 2:** Type III ANOVA results for main effects and interactions on back beliefs (Back‐PAQ10), lumbar spine motion (lumbar curvature RoM), and lifting strategy (Stoop–Squat Index).

Effect	Back‐PAQ10		Lumbar curvature RoM		Stoop‐squat index	
	F (df1, df2)	*p*‐value	F (df1, df2)	*p*‐value	F (df1, df2)	*p*‐value
Group	F (1, 44) = 5.62	0.022	F (1, 46.58) = 1.01	0.320	F (1, 44.54) = 4.10	0.049
Time	F (1, 348) = 308.34	< 0.001	F (1, 244.00) = 39.61	< 0.001	F (1, 244.00) = 8.05	0.005
Task	—	—	F (1, 48.00) = 154.65	< 0.001	F (1, 48.00) = 30.48	< 0.001
Load	—	—	F (1, 48.00) = 12.06	0.001	F (1, 48.00) = 7.83	0.007
Group × time	F (1, 348) = 172.78	< 0.001	F (1, 244.00) = 0.27	0.604	F (1, 244.00) = 0.01	0.935
Group × task	—	—	F (1, 68.37) = 0.18	0.673	F (1, 71.25) = 0.09	0.766
Group × load	—	—	F (1, 114.98) = 2.33	0.130	F (1, 69.66) = 1.96	0.166
Group × time × task	—	—	F (2, 244.00) = 0.28	0.759	F (2, 244.00) = 0.18	0.835
Group × time × load	—	—	F (2, 244.00) = 0.13	0.881	F (2, 244.00) = 0.57	0.564

**FIGURE 3:**
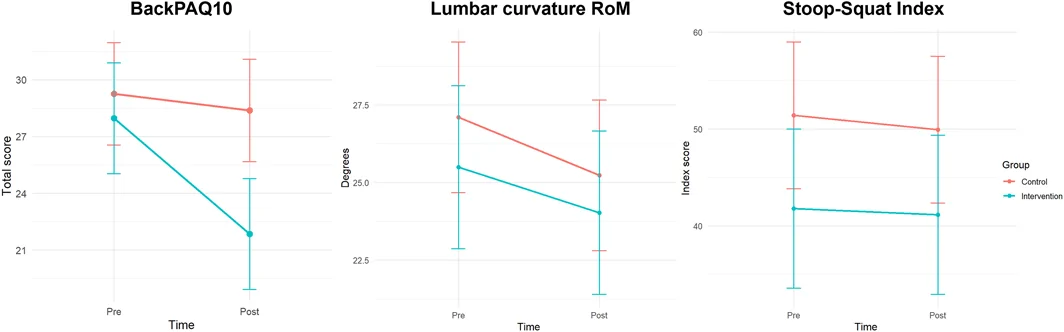
Effects of the interventions on back beliefs (Back‐PAQ10; left), lumbar spine motion (lumbar curvature RoM; middle), and lifting strategy (Stoop‐Squat Index; right), averaged across Task (lifting and lowering) and Load (5 and 15 kg) conditions. Values are presented as means with 95% confidence intervals.

#### 3.2.2 Lumbar Spine Motion and Lifting Strategy

For lumbar curvature RoM, the model showed no Group × Time interaction (*p* = 0.604), but a significant main effect of Time (*p* < 0.001) (Table 2), indicating a change over time that was similar in the experimental and control groups. Task (*p* < 0.001) and Load (*p* = 0.001) also showed significant main effects. However, no significant Group × Time × Task (*p* = 0.759) or Group × Time × Load (*p* = 0.881) interactions were observed, indicating that the interventions did not differentially affect lumbar curvature RoM across tasks or loads. Estimated marginal means showed that lumbar curvature RoM decreased significantly from Pre to Post in both groups (Figure 3). This decrease was consistently observed within each Task (lifting and lowering) and Load (5 and 15 kg) condition when analysed separately (Figure 4).

**FIGURE 4:**
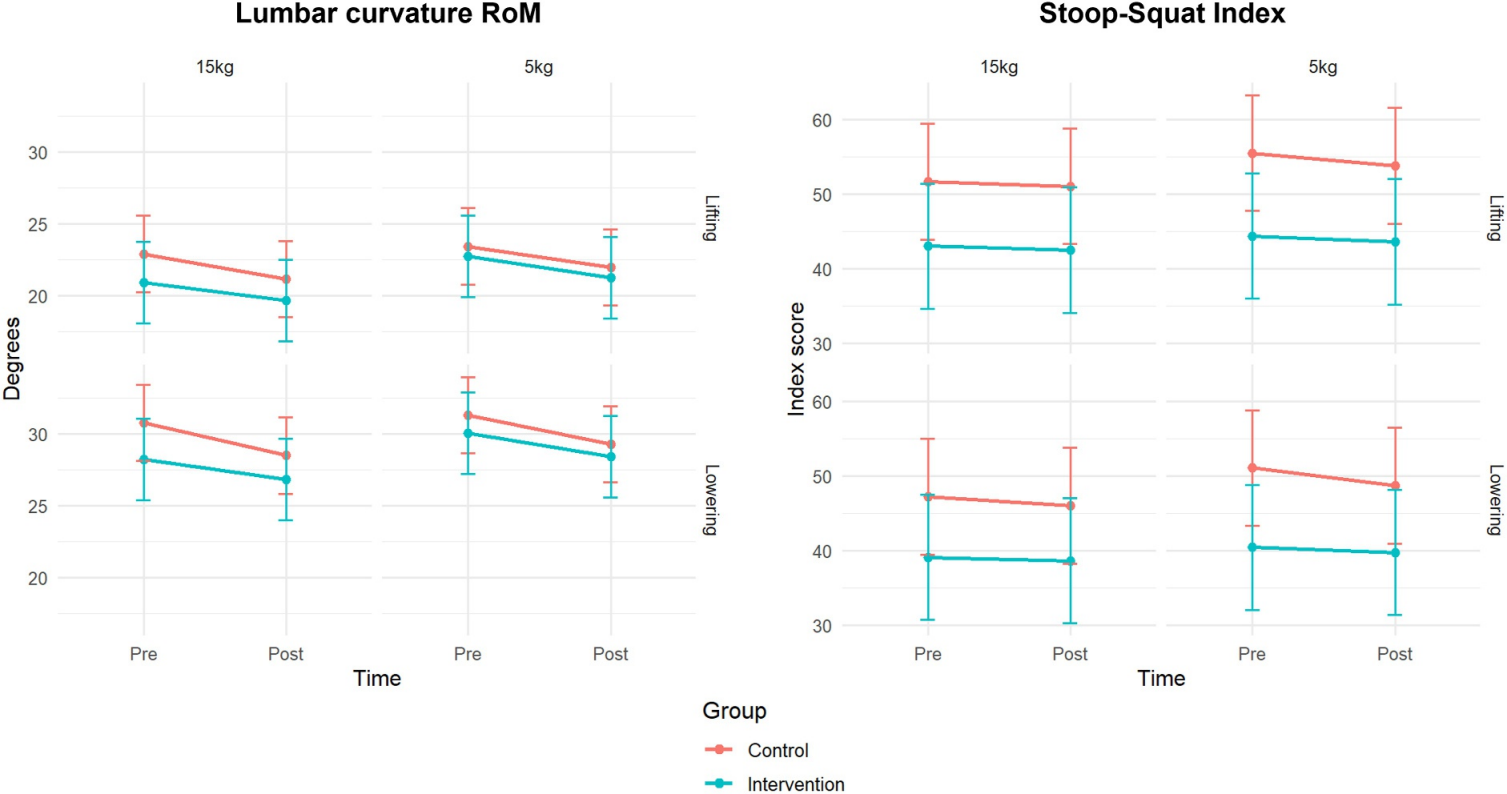
Effects of the interventions on lumbar spine motion (lumbar curvature RoM; left) and lifting strategy (Stoop‐Squat Index; right), stratified by Task (lifting and lowering) and Load (5 and 15 kg). Values are presented as means with 95% confidence intervals.

For the Stoop‐Squat Index, no significant Group × Time interaction was observed (*p* = 0.935) (Table 2), indicating that changes over time did not differ between the experimental and control groups. Significant main effects of Time (*p* = 0.005) and Group (*p* = 0.049) were found, indicating an overall change from Pre to Post as well as overall differences between the groups. No significant Group × Time × Task (*p* = 0.835) or Group × Time × Load (*p* = 0.564) interactions were observed, suggesting that the intervention effects did not vary across tasks or loads. In contrast, significant main effects of Task (*p* < 0.001) and Load (*p* = 0.007) indicated overall differences between lifting and lowering and between the 5 and 15 kg conditions. Estimated marginal means indicated a significant decrease in the Stoop‐Squat Index from Pre to Post in the control group, whereas no significant change was observed in the experimental group (Figure 3). When analysed separately, this decrease was primarily observed during the lowering task and under the 5 kg load condition in the control group (Figure 4), although no significant interactions with Task or Load were found.

## 4 Discussion

The aim of the present study was to determine whether a brief evidence‐based, belief‐focused educational video could reduce unhelpful beliefs about back pain and promote a more stoop‐like behaviour during object lifting in pain‐free adults. The results indicate a dissociation between cognitive and behavioural outcomes. While the evidence‐based, belief‐focused education led to a substantial improvement in back pain beliefs, it did not result in measurable changes in lifting behaviour.

With respect to back pain beliefs, the evidence‐based, belief‐focused education appeared to be effective. Back‐PAQ10 scores improved more in the experimental group than in the control group. This finding suggests that a short, 3‐min evidence‐based, belief‐focused educational video may modify unhelpful beliefs about back pain in the short term. This is in line with previous research showing that brief, targeted educational interventions can positively influence health‐related beliefs. For example, Darlow et al. (2013) demonstrated that clinicians' explanations of back pain can have a lasting impact on patients' beliefs and behaviours, with more negative or biomedical messages associated with increased fear and unhelpful beliefs. Egerton et al. (2021) reported that short educational video improved patients' understanding and management of knee osteoarthritis. In a broader context, systematic reviews have shown that mass media and educational campaigns can effectively shift public beliefs about LBP (Grilli et al. 2002; Suman et al. 2021). The effectiveness of such interventions appears to depend strongly on the framing of the message, with content emphasising positive outcomes and incorporating emotion‐based elements, which have been shown to enhance message effectiveness in public health campaigns (Kim 2015). In the present study, the evidence‐based, belief‐focused educational video incorporated these elements by featuring healthcare professionals and an individual with lived experience, highlighting the benefits of movement and discouraging catastrophic interpretations of back pain, which likely contributed to the observed improvements.

In contrast, no intervention‐specific effects were observed for biomechanical outcomes. Lumbar curvature RoM decreased from pre to post in both groups, with consistent reductions across all task (lifting and lowering) and load (5 and 15 kg) conditions, and no interaction between time and group. A similar pattern was observed for the Stoop‐Squat Index, with participants tending to adopt a more squat‐like lifting strategy over time, while the experimental group generally exhibited a slightly more pronounced squat strategy. These findings suggest that the observed changes over time are unlikely attributable to the interventions and may instead reflect other factors.

The lack of behavioural change despite reduced negative back beliefs highlights the gap between cognitive factors and motor behaviour. Lifting strategies are likely shaped by entrenched motor patterns, physical capacity, and contextual constraints. Movement patterns are expected to be well established in the study population, given the mean age of approximately 40 years. Moreover, traditional ergonomic advice and many occupational manual‐handling guidelines continue to favour squat or semi‐squat lifting and limiting lumbar flexion, potentially reinforcing the perception that bending the back is dangerous (Darlow et al. 2013; Health and Safety Executive 2025; National Institutes of Health. n.d.; Nolan et al. 2018; Suva n.d.). Consequently, a single 3‐min evidence‐based, belief‐focused educational video is likely insufficient to override these long‐standing habitual motor patterns. More intensive or interactive interventions, potentially incorporating practice, feedback, or discussion, may be required to induce behavioural change. Such approaches could combine cognitive components (e.g., addressing maladaptive beliefs) with behavioural strategies, such as gradual exposure to feared movements (Krug et al. 2022), as exemplified by Cognitive Functional Therapy (O’Sullivan et al. 2018). This interpretation is consistent with evidence that advice or education produces only small short‐term improvements in pain and disability when delivered alone to people with non‐specific spinal pain, suggesting that education may have greater effects when integrated with other interventions (Jones et al. 2021).

The observed main effects for Time may also reflect repeated‐task effects, including learning, pacing adjustments, or neuromuscular fatigue. Fatigue can be task dependent and may be more pronounced during unfamiliar movements (Enoka 1995). Given the sedentary nature of participants' occupations, the repeated lifting trials‐particularly with a higher load‐may have induced local or whole‐body neuromuscular fatigue and subtle changes in movement strategy. Because fatigue was not measured, however, this explanation remains speculative. Additionally, limited trunk and hamstring flexibility may have constrained the adoption of a more stoop‐like strategy. This may have been particularly relevant for men, who typically display less flexibility than women (Wydra and Heckmann 2007). Consistent with this possibility, fingertip‐to‐floor distance was descriptively greater in men than in women in the present sample (7.3 ± 9.1 cm vs. 0.4 ± 1.1 cm, respectively), indicating lower combined trunk and hamstring flexibility. However, because the relationship between flexibility and lifting strategy was not specifically examined, this interpretation remains speculative.

This study has several strengths. Its randomised controlled design, complete follow‐up, comparable baseline characteristics, and mixed‐effects analyses across tasks and loads support the internal consistency of the findings. Moreover, data collection at the participants' workplace represents a real‐life setting and may better reflect the immediate effects of brief education as it could be delivered outside a clinical or laboratory environment. Several limitations should nevertheless be acknowledged. The experimental intervention was brief and not reinforced (i.e., no follow‐up instructions or practical sessions), which may have limited its effect on behaviour. In addition, the short time frame between the intervention and re‐assessment does not allow conclusions regarding the sustainability of the observed changes in back pain beliefs. Moreover, although measurements were conducted in a real‐world setting, the standardised setup, including the use of the Epionics SPINE system, may have constrained participants' natural movement patterns. Repeated execution of the lifting task could have introduced fatigue or learning effects. In addition, participants working in the same environment may have exchanged information, potentially influencing outcomes. Furthermore, the inclusion of only pain‐free individuals limits the generalisability of these findings to clinical populations. Finally, because the feasibility‐based sample size was not informed by a formal power calculation, the study may have lacked precision to detect smaller effects, particularly for biomechanical outcomes. The estimates should be interpreted as exploratory and used to plan adequately powered confirmatory trials.

### 4.1 Future Research

Future research should examine whether repeated or reinforced evidence‐based education produces sustained changes in back beliefs and whether combining education with movement practice, feedback, graded exposure, or other interventions facilitates behavioural change. Studies should include longer follow‐up, adequately powered samples, direct assessment of intervention mechanisms and fatigue, and populations with LBP or physically demanding work. They should also test which message components, including lived‐experience narratives, are most effective.

### 4.2 Conclusion

A single 3‐min evidence‐based, belief‐focused educational video produced a larger immediate reduction in unhelpful back beliefs than a structure‐focused anatomy video in pain‐free office workers. It did not, however, alter lumbar spine motion or overall lifting strategy when participants handled 5 and 15 kg boxes, highlighting the dissociation between cognitive and motor responses. Brief evidence‐based, belief‐focused educational videos may therefore be useful for modifying beliefs, whereas changing entrenched motor behaviour is likely to require more intensive or interactive approaches.

## Author Contributions

**Anja Müller‐Schmid:** conceptualisation, methodology, investigation, data curation, formal analysis, writing – original draft. **Christian Bangerter:** methodology, data curation, formal analysis, software, validation, writing – review and editing. **Panka Nagy:** conceptualisation, methodology, writing – review and editing. **Fabian Pfeiffer:** methodology, resources, writing – review and editing. **Marina Bruderer‐Hofstetter:** methodology, resources, writing – review and editing. **Guillaume Christe:** conceptualisation, methodology, supervision, writing – review and editing. **Stefan Schmid:** conceptualisation, methodology, formal analysis, software, validation, visualisation, resources, project administration, supervision, writing – original draft.

## Funding

The authors have nothing to report.

## Ethics Statement

Ethical approval for this study was obtained from the ethics committee of the canton of Bern in Switzerland (KEK Bern; project ID: 2023–01182, approval date: 21 July 2023, PI/Sponsor: Stefan Schmid). The study was conducted in accordance with the ethical principles of the Declaration of Helsinki and Good Clinical Practice (GCP) guidelines.

## Conflicts of Interest

One author (G.C.) was involved in the development of the French version of the evidence‐based, belief‐focused educational video used as experimental intervention in this study and co‐authored the publication by Corbat et al. (2020). This work had no commercial purpose, and the authors declare no financial or commercial conflicts of interest.

## Supporting information


Supporting Information S1

Supporting Information S2

## Data Availability

The data that support the findings of this study are available from the corresponding author upon reasonable request.

## References

[msc70290-bib-0001] Au, I. P. H. , N. Saraceni , A. Smith , P. O’Sullivan , L. Ng , and A. Campbell . 2025. “Is Lifting Technique Related to Pain and Functional Limitation? A Replicated Single‐Case Design Study of Five People With Lifting‐Related Chronic Low Back Pain.” European Journal of Pain 29, no. 6: e70071. 10.1002/ejp.70071. PMC12214296 40596778

[msc70290-bib-0002] Bangerter, C. , O. Faude , P. Eichelberger , et al. 2024. “Conventional Video Recordings Dependably Quantify Whole‐Body Lifting Strategy Using the Stoop‐Squat‐Index: A Methods Comparison Against Motion Capture and a Reliability Study.” Journal of Biomechanics 164: 111975. 10.1016/j.jbiomech.2024.111975. 38320342

[msc70290-bib-0003] Bangerter, C. , O. Faude , D. Weidinger , et al. 2025. “Task‐Specific Pain‐Related Fear Influences Lifting Biomechanics Differently in Individuals With and Without Occupations Involving Repetitive Lifting Tasks.” Scientific Reports 15, no. 1: 41098. 10.1038/s41598-025-24890-z. PMC12635262 41266444

[msc70290-bib-0004] Belavy, D. L. , M. Quittner , N. D. Ridgers , et al. 2019. “Beneficial Intervertebral Disc and Muscle Adaptations in High‐Volume Road Cyclists.” Medicine and Science in Sports and Exercise 51, no. 1: 211–217. 10.1249/MSS.0000000000001770. 30157104

[msc70290-bib-0005] Chang, J. R. , C. G. Maher , S. Sharma , et al. 2026. “What do the General Public Believe About Back Pain? A Scoping Review of Population‐Based Studies.” BMC Public Health 26, no. 1: 1158. 10.1186/s12889-026-26879-3. PMC13064184 41776493

[msc70290-bib-0006] Christe, G. , V. Pizzolato , M. Meyer , J. Nzamba , and C. Pichonnaz . 2021. “Unhelpful Beliefs and Attitudes About Low Back Pain in the General Population: A Cross‐Sectional Survey.” Musculoskeletal Science & Practice 52: 102342. 10.1016/j.msksp.2021.102342. 33582619

[msc70290-bib-0007] Corbat, D. , P. Montavon , and G. Christe . 2020. “Développement et évaluation du site d’information sur la lombalgie: Www.Infomaldedos.Ch.” Mains Libres 37, no. 2: 79–87. 10.55498/mainslibres.2020.37.2.0079.

[msc70290-bib-0008] Darlow, B . 2016. “Beliefs About Back Pain: The Confluence of Client, Clinician and Community.” International Journal of Osteopathic Medicine 20: 53–61. 10.1016/j.ijosm.2016.01.005.

[msc70290-bib-0009] Darlow, B. , A. Dowell , G. D. Baxter , F. Mathieson , M. Perry , and S. Dean . 2013. “The Enduring Impact of What Clinicians Say to People With Low Back Pain.” Annals of Family Medicine 11, no. 6: 527–534. 10.1370/afm.1518. PMC3823723 24218376

[msc70290-bib-0010] Darlow, B. , M. Perry , F. Mathieson , et al. 2014. “The Development and Exploratory Analysis of the Back Pain Attitudes Questionnaire (Back‐PAQ).” BMJ Open 4, no. 5: e005251. 10.1136/bmjopen-2014-005251. PMC4039861 24860003

[msc70290-bib-0011] Deyo, R. A. , S. K. Mirza , J. A. Turner , and B. I. Martin . 2009. “Overtreating Chronic Back Pain: Time to Back Off?” Journal of the American Board of Family Medicine: JABFM 22, no. 1: 62–68. 10.3122/jabfm.2009.01.080102. PMC2729142 19124635

[msc70290-bib-0012] Egerton, T. , L. McLachlan , B. Graham , et al. 2021. “How Do People With Knee Pain From Osteoarthritis Respond to a Brief Video Delivering Empowering Education About the Condition and its Management?” Patient Education and Counseling 104, no. 8: 2018–2027. 10.1016/j.pec.2021.01.007. 33531156

[msc70290-bib-0013] Enoka, R. M . 1995. “Mechanisms of Muscle Fatigue: Central Factors and Task Dependency.” Journal of Electromyography and Kinesiology Official Journal of the International Society of Electrophysiological Kinesiology 5, no. 3: 141–149. 10.1016/1050-6411(95)00010-w. 20719645

[msc70290-bib-0014] Foss, I. S. , I. Holme , and R. Bahr . 2012. “The Prevalence of Low Back Pain Among Former Elite Cross‐Country Skiers, Rowers, Orienteerers, and Nonathletes: A 10‐Year Cohort Study.” American Journal of Sports Medicine 40, no. 11: 2610–2616. 10.1177/0363546512458413. 22972850

[msc70290-bib-0015] Grilli, R. , C. Ramsay , and S. Minozzi . 2002. “Mass Media Interventions: Effects on Health Services Utilisation.” Cochrane Database of Systematic Reviews 2010, no. 1: CD000389. 10.1002/14651858.CD000389. PMC13429285 11869574

[msc70290-bib-0016] Hartvigsen, J. , M. J. Hancock , A. Kongsted , et al. 2018. “What Low Back Pain is and Why we Need to Pay Attention.” Lancet 391, no. 10137: 2356–2367. 10.1016/S0140-6736(18)30480-X. 29573870

[msc70290-bib-0017] Health and Safety Executive . 2025. “Manual Handling at Work: Good Handling Technique.” Health and Safety Executive. https://www.hse.gov.uk/msd/manual‐handling/good‐handling‐technique.htm.

[msc70290-bib-0018] Jones, C. M. , C. A. Shaheed , G. E. Ferreira , P. Kharel , C.‐W. Christine Lin , and C. G. Maher . 2021. “Advice and Education Provide Small Short‐Term Improvements in Pain and Disability in People With Non‐Specific Spinal Pain: A Systematic Review.” Journal of Physiotherapy 67, no. 4: 263–270. 10.1016/j.jphys.2021.08.014. 34518145

[msc70290-bib-0019] Kim, H. S . 2015. “Attracting Views and Going Viral: How Message Features and News‐Sharing Channels Affect Health News Diffusion.” Journal of Communication 65, no. 3: 512–534. 10.1111/jcom.12160. PMC4591750 26441472

[msc70290-bib-0020] Knechtle, D. , S. Schmid , M. Suter , et al. 2020. “Fear‐Avoidance Beliefs are Associated With Reduced Lumbar Spine Flexion During Object Lifting in Pain‐Free Adults.” Pain 162, no. 6: 1621–1631. 10.1097/j.pain.0000000000002170. PMC8120682 33323888

[msc70290-bib-0021] Krug, R. C. , M. F. Silva , O. V. Lipp , et al. 2022. “An Investigation of Implicit Bias About Bending and Lifting.” Scandinavian Journal of Pain 22, no. 2: 336–347. 10.1515/sjpain-2021-0145. 34821139

[msc70290-bib-0022] Lagersted‐Olsen, J. , B. L. Thomsen , A. Holtermann , K. Søgaard , and M. B. Jørgensen . 2016. “Does Objectively Measured Daily Duration of Forward Bending Predict Development and Aggravation of Low‐Back Pain? A Prospective Study.” Scandinavian Journal of Work, Environment and Health 42, no. 6: 528–537. 10.5271/sjweh.3591. 27606607

[msc70290-bib-0023] Maher, C. , M. Underwood , and R. Buchbinder . 2017. “Non‐Specific Low Back Pain.” Lancet 389, no. 10070: 736–747. 10.1016/S0140-6736(16)30970-9. 27745712

[msc70290-bib-0024] Maillard, A. , T. Pasche , P. Coenen , and G. Christe . 2026. “The Association Between Trunk Flexion and Low Back Pain in Blue‐Collar Workers: A Systematic Review.” Work 84, no. 1: 42–51. 10.1177/10519815251397391. PMC13144654 41359499

[msc70290-bib-0025] Mohler, M. , D. Nyfeler , M. Bruderer‐Hofstetter , et al. Construct Validity of the German Version of the Back Pain Attitudes Questionnaire (Back‐PAQ‐G): (Under Review).

[msc70290-bib-0026] Mueller, A . 2020. Rückenreport Schweiz 2020. Rheumaliga Schweiz. https://www.rheumaliga.ch/news/rueckenreport‐2020.html.

[msc70290-bib-0027] National Guideline Centre (UK) . 2016. Low Back Pain and Sciatica in over 16s: Assessment and Management. National Institute for Health and Care Excellence (NICE). http://www.ncbi.nlm.nih.gov/books/NBK401577/. 27929617

[msc70290-bib-0028] National Institutes of Health . n.d. Back Health. Office of Research Services. Accessed September 10, 2026. https://ors.od.nih.gov/sr/dohs/HealthAndWellness/Ergonomics/Pages/spine.aspx.

[msc70290-bib-0029] Nolan, D. , K. O’Sullivan , J. Stephenson , P. O’Sullivan , and M. Lucock . 2018. “What do Physiotherapists and Manual Handling Advisors Consider the Safest Lifting Posture, and do Back Beliefs Influence Their Choice?” Musculoskeletal Science and Practice 33: 35–40. 10.1016/j.msksp.2017.10.010. 29078081

[msc70290-bib-0030] Nyfeler, D. , M. Mohler , F. Pfeiffer , et al. 2026. “Cross‐Cultural Adaptation and Reliability Investigation of the German Version of the Back Pain Attitudes Questionnaire (Back‐PAQ‐G).” BMC Musculoskeletal Disorders. 10.1186/s12891-026-10584-8.

[msc70290-bib-0031] Nzamba, J. , S. Van Damme , J. Favre , and G. Christe . 2024. “The Relationships Between Spinal Amplitude of Movement, Pain and Disability in Low Back Pain: A Systematic Review and Meta‐Analysis.” European Journal of Pain 28, no. 1: 37–53. 10.1002/ejp.2162. 37475698

[msc70290-bib-0032] O’Keeffe, M. , C. G. Maher , T. R. Stanton , et al. 2019. “Mass Media Campaigns are Needed to Counter Misconceptions About Back Pain and Promote Higher Value Care.” British Journal of Sports Medicine 53, no. 20: 1261–1262. 10.1136/bjsports-2018-099691. 30377173

[msc70290-bib-0033] O’Sullivan, P . 2012. “It’s Time for Change With the Management of Non‐Specific Chronic Low Back Pain.” British Journal of Sports Medicine 46, no. 4: 224–227. 10.1136/bjsm.2010.081638. 21821612

[msc70290-bib-0034] O’Sullivan, P. , J. P. Caneiro , M. O’Keeffe , et al. 2018. “Cognitive Functional Therapy: An Integrated Behavioral Approach for the Targeted Management of Disabling Low Back Pain.” Physical Therapy 98, no. 5: 408–423. 10.1093/ptj/pzy022. PMC6037069 29669082

[msc70290-bib-0035] O’Sullivan, P. , and I. Lin . 2014. “Acute Low Back Pain: Beyond Drug Therapies.” Pain Management Today 1, no. 1: 8–13. https://painmanagement.medicinetoday.com.au/system/files/pdf/pain_management_today/article/PMT2014‐01‐008‐O%27SULLIVAN.pdf.

[msc70290-bib-0036] Perret, C. , S. Poiraudeau , J. Fermanian , M. M. Colau , M. A. Benhamou , and M. Revel . 2001. “Validity, Reliability, and Responsiveness of the Fingertip‐To‐Floor Test.” Archives of Physical Medicine and Rehabilitation 82, no. 11: 1566–1570. 10.1053/apmr.2001.26064. 11689977

[msc70290-bib-0037] Pierobon, A. , and B. Darlow . 2023. “Back Pain Attitudes Questionnaire (Back‐PAQ).” In International Handbook of Behavioral Health Assessment. Springer.

[msc70290-bib-0038] Saraceni, N. , P. Kent , L. Ng , A. Campbell , L. Straker , and P. O’Sullivan . 2020. “To Flex or Not to Flex? Is There a Relationship Between Lumbar Spine Flexion During Lifting and Low Back Pain? A Systematic Review With Meta‐Analysis.” Journal of Orthopaedic and Sports Physical Therapy 50, no. 3: 121–130. 10.2519/jospt.2020.9218. 31775556

[msc70290-bib-0039] Schmid, S . 2022. “The Stoop‐Squat‐Index: A Simple but Powerful Measure for Quantifying Whole‐Body Lifting Behavior.” Archives of Physiotherapy 12, no. 1: 8. 10.1186/s40945-022-00135-4. PMC9027351 35449120

[msc70290-bib-0040] Suman, A. , S. Armijo‐Olivo , S. Deshpande , et al. 2021. “A Systematic Review of the Effectiveness of Mass Media Campaigns for the Management of Low Back Pain.” Disability and Rehabilitation 43, no. 24: 3523–3551. 10.1080/09638288.2020.1743777. 32242464

[msc70290-bib-0041] Suter, M. , P. Eichelberger , J. Frangi , E. Simonet , H. Baur , and S. Schmid . 2020. “Measuring Lumbar Back Motion During Functional Activities Using a Portable Strain Gauge Sensor‐Based System: A Comparative Evaluation and Reliability Study.” Journal of Biomechanics 100: 109593. 10.1016/j.jbiomech.2019.109593. 31898974

[msc70290-bib-0042] Swiss National Accident Insurance Fund (Suva) . n.d. Lasten clever anpacken. Suva. Accessed September 10, 2026. https://www.suva.ch/de‐ch/praevention/nach‐gefahren/gefaehrliche‐materialien‐strahlungen‐und‐situationen/koerperliche‐belastung‐und‐ergonomie/umgang‐mit‐schweren‐lasten/heben‐sie‐lasten‐clever.

[msc70290-bib-0043] Wernli, K. , A. Smith , F. Coll , A. Campbell , P. Kent , and P. O’Sullivan . 2022. “From Protection to Non‐Protection: A Mixed Methods Study Investigating Movement, Posture and Recovery From Disabling Low Back Pain.” European Journal of Pain 26, no. 10: 2097–2119. 10.1002/ejp.2022. PMC9826080 35959703

[msc70290-bib-0044] Wydra, G. , and M. Heckmann . 2007. “Discrepancies of Mobility in Dependence on Anatomical Sidedness Differences.” Deutsche Zeitschrift für Sportmedizin 58, no. 11: 401–405. https://www.germanjournalsportsmedicine.com/fileadmin/content/archiv2007/heft11/401‐405.pdf.

